# Journal of hematology & oncology: A journal open to all

**DOI:** 10.1186/1756-8722-1-1

**Published:** 2008-05-28

**Authors:** Delong Liu

**Affiliations:** 1 Associate Professor of Medicine, New York Medical College, Munger Pavilion 250, Valhalla, NY 10595, USA

## Editorial

Paul Ehrlich prophesied "magic bullet" in early 1900 [1], but it was not until 1975 that Kohler and Milstein developed the hybridoma technology [2]. Since then hematology and oncology have become closely intertwined and rapidly evolving fields. New findings from laboratory-based research are rapidly being turned into clinical applications. Within less than a decade, targeted therapy has become a treatment of choice for many diseases, with imatinib and rituximab representing the many recent major breakthroughs in cancer therapy [3,4].

The growth of the internet has shrunk the world to a mouse click, yet the dissemination of new scientific findings is still very restricted. As a scientist and a clinician, I have to squeeze out time to publish new scientific findings and clinical observations. It almost always takes months to get a manuscript published. Frequently, the published articles are accessible only to those with subscriptions. *Journal of Hematology & Oncology *aims to serve as an international platform for sharing laboratory and clinical findings in an open access format among laboratory scientists, physician scientists, hematologists and oncologists. A rapid turnaround time from submission to publication means that knowledge and new successes can be shared in real time.

Rapid advances in molecular biology and the completion of genome mapping from "worm to man" have led to an explosive increase in discoveries of new genes and targets, which result in development of new drugs and new technologies for diagnosis and therapy of medical disorders, especially for blood and cancer diseases. Studies of tyrosine kinase oncogenes and signal transductions paved the way to the discoveries of tyrosine kinase inhibitors [5]. The "magic bullet" theory has finally become a reality in cancer therapy with the advent of novel drugs, such as gemtuzumab ozogamycin (mylotarg), denileukin diftitox (ontak), tositumomab (bexxar), and ibritumomab (zevalin), to name a few. Studies of angiogenesis and monoclonal antibodies make it possible for a new modality of cancer therapy [6]. Not until recently, ubiquitin and heat-shock proteins were only familiar to scientists who are "mouse doctors", but not to "human doctors". However, proteasome inhibitors that target the ubiquinization-pathway have virtually revolutionized therapies for multiple myeloma in only a few years [7]. Epigenetic studies have led to the development of new drugs which have changed the lives of patients with myelodysplastic syndrome [8]. Clinical trials are increasingly performed in many centers across the world.

With all these rapid developments and findings, volume of information has grown enormously. However, many publications are in inaccessible places, and many more are not published until months later or not at all. A few top-rated journals in the field of hematology and oncology already exist. Many doctors and scientists from developing countries can not afford to pay for the access to these expensive journals. Meanwhile, journals become more focused and increasingly specialized.

*Journal of Hematology & Oncology *aims not to specialize, rather to broaden and provide a platform for information exchange for all studies related to blood and cancer. It aims to include, not to exclude, all studies from basic research, translational research, case reports, and clinical trials. This journal allows the authors to keep the copyright so they can freely use and disseminate their articles as they please. All articles published in this journal are also archived in PubMed, PubMed Central, and other repositories. Therefore, this journal aims not to restrict, rather to make all published articles free and open to all.

## References

[B1] Stone MJ (2006). Monoclonal Antibodies-designer medical missiles. Lancet.

[B2] Köhler G, Milstein C (1975). Continuous cultures of fused cells secreting antibody of predefined specificity. Nature.

[B3] Druker BJ, Tamura S, Buchdunger E, Ohno S, Segal GM, Fanning S, Zimmermann J, Lydon NB Effects of a selective inhibitor of the Abl tyrosine kinase on the growth of Bcr-Abl positive cells. Nat Med.

[B4] Vose JM, Link BK, Grossbard ML, Czuczman M, Grillo-Lopez A, Gilman P, Lowe A, Kunkel LA, Fisher RI (2001). Phase II Study of Rituximab in Combination With CHOP Chemotherapy in Patients With Previously Untreated, Aggressive Non-Hodgkin's Lymphoma. J Clin Oncol.

[B5] Liu D, Wang L-H (1994). Oncogenes, Protein Tyrosine Kinases, and Signal Transduction. J Biomed Sci.

[B6] Folkman J (1971). Tumor angiogenesis: therapeutic implications. New England Journal of Medicine.

[B7] Kyle RA, Rajkumar SV (2004). Multiple Myeloma. NEJM.

[B8] Silverman LR, Demakos EP, Peterson BL, Kornblith AB, Holland JC, Odchimar-Reissig R, Stone RM, Nelson D, Powell BL, DeCastro CM, Ellerton J, Larson RA, Schiffer CA, Holland J (2002). Randomized controlled trial of azacitidine in patients with the myelodysplastic syndrome: a study of the Cancer and Leukemia Group B. J Clin Oncol.

